# Trends in the annual incidence and risk factors of cerebral palsy

**DOI:** 10.1038/s41390-025-04752-0

**Published:** 2026-01-23

**Authors:** Jooyoung Lee, Yoonjeong Choi, Miji Kim, Woo Hyung Lee, Ja-Ho Leigh, Moon Suk Bang

**Affiliations:** 1 Department of Pediatrics, Eunpyeong St. Mary’s Hospital, College of Medicine, The Catholic University of Korea, Seoul, Republic of Korea; 2 Department of Rehabilitation Medicine, Seoul National University Hospital, Seoul, Republic of Korea; 3 National Traffic Injury Rehabilitation Research Institute, National Traffic Injury Rehabilitation Hospital, Yangpyeong, Republic of Korea; 4 Department of Rehabilitation Medicine, Seoul National University College of Medicine, Seoul, Republic of Korea

## Abstract

**Background:**

Cerebral palsy (CP) arises from non-progressive brain damage occurring during the prenatal, perinatal, or postnatal period. We analyzed CP incidence and risk factors in South Korea over a 20-year period.

**Methods:**

We included children newly diagnosed with CP between the first year of life and 5 years from 2005 to 2024 and examined their CP risk factors. Annual incidence rates were calculated by dividing the number of new cases each year by the population of children aged ≤5 years, with age- and sex-standardization. The CP cumulative incidence at the 5-year follow-up was analyzed by birth year. Temporal incidence trends were assessed using Joinpoint regression.

**Results:**

The annual age- and sex-standardized CP incidence rate decreased from 6.6 to 3.6/10,000 between 2005 and 2024. The 5-year cumulative CP incidence also declined from 4.26/1000 live births in 2005 to 2.51/1000 live births in 2020. The most prevalent risk factors were birth-related complications, prematurity/low birth weight, and metabolic diseases, all showing increasing trends.

**Conclusion:**

From 2005 to 2024, the CP incidence in South Korea declined, possibly reflecting perinatal-care improvements, particularly among children aged ≤2 years. Nationwide developmental-screening programs may have also enhanced milder-case detection, perhaps increasing the relative proportion diagnosed at ages 4–5 years.

**Impact:**

- Over the past 20 years, the annual incidence of cerebral palsy diagnosis in children between the first year of life and 5 years old has gradually decreased in South Korea.
- Cerebral palsy was the most frequently diagnosed at 1 year of age, and birth-related conditions, prematurity or low birth weight, and metabolic diseases were the most prevalent risk factors showing an increasing trend.
- This study suggests that advances in perinatal care might have contributed to the decline in CP incidence and the developmental screening test for infants and children implemented in South Korea may have contributed to reduction of missed diagnoses.

## Introduction

Cerebral palsy (CP) is comprised of a group of disorders characterized by permanent movement, postural abnormalities, and activity limitations. It is the most common cause of movement disorders in children,^1^ and can be accompanied by various neurodevelopmental problems, such as intellectual disability, autism, epilepsy, visual/hearing impairment, cognitive impairment, and physical problems, such as pain, hip displacement, and incontinence.^2^ In addition, the socioeconomic burden of CP is significant.^3,4^ CP results from non-progressive damage that occurs during development of the immature brain. These brain injuries occur during the prenatal, perinatal, and postnatal periods due to various causes.^5,6^ Prenatal and perinatal causes account for the majority of CP, with postnatal causes accounting for approximately 10%.^2^ The best-known risk factors for CP are prematurity and low birth weight, with shorter gestational age and lower birth weight associated with an increased incidence of CP.^7^ According to a previous meta-analysis of population-based studies mainly from high-income countries, the prevalence of CP has remained unchanged at 2–2.5 per 1000 live births between 2006 and 2010, and the global pooled prevalence of CP was 2.11 per 1000 live births.^7^ However, the prevalence of CP varies according to economic level, with high-income countries having a lower prevalence than low- or middle-income countries. A recent study reported that the prevalence of CP in high-income countries is gradually declining, widening the gap between low- and middle-income countries.^8^

The prevalence of CP in South Korea from 2004 to 2008 was 2.6 per 1000 children,^9^ which is higher than the global pooled prevalence. The 4-year incidence of CP in South Korea declined from 4.77 to 2.52 per 1,000 live births between 2007 and 2015.^10^ Another previous study in South Korea reported that the proportion of neonates diagnosed with CP decreased from 0.41% in 2007 to 0.25% in 2011.^11^ The gradual decline in the incidence of CP in Korea is similar to high-income countries, despite a gradual increase in high-risk births due to older pregnancies. However, these studies have identified short-term trends of 5 years or less.^10,11^ A previous study based on national registry data from 2013 to 2014 has confirmed an increase in the incidence of CP due to prematurity and low birth weight in South Korea,^12^ comprehensive analyses of the underlying risk factors were not conducted.

The objectives of this study were to: (1) determine the incidence of CP in Korea and analyze trends over the past 20 years using nationwide insurance claims data from the National Health Insurance Service (NHIS), (2) compare these trends with international annual incidence rates and prevalence, and (3) examine the distribution and temporal changes of risk factors for CP.

## Methods

### Data source and study population

We assessed the incidence of pediatric CP in South Korea using data from the NHIS between 2005 and 2024. South Korea operates a mandatory single-payer healthcare system, where all medical institutions—ranging from primary clinics to tertiary hospitals—submit claims to a single data repository managed by the NHIS.^13^ This comprehensive claims system includes diagnostic records from all types of healthcare providers, including general practitioners and pediatricians, across both inpatient and outpatient settings.^14^ As such, the NHIS data capture virtually all clinically diagnosed cases of CP at the national level, enabling comprehensive population-based identification of CP cases across the entire healthcare spectrum.

Patients with pediatric CP were identified according to the International Classification of Diseases, 10th Revision (ICD-10): cerebral palsy (G80) and its subtypes: spastic cerebral palsy (G80.0), dyskinetic cerebral palsy (G80.3), ataxic cerebral palsy (G80.4), other cerebral palsy (G80.8), and unspecified cerebral palsy (G80.9). Between 2005 and 2024, patients with primary or secondary diagnosis code of CP at least twice within 5 years after birth were included in the retrospective CP cohort. After a washout of patients diagnosed with CP between 1999 and 2004, only new cases of pediatric CP in each year from 2005 to 2024 were selected as the final study population.

The year of CP onset was defined as the earliest year in which a child received a CP diagnosis following their birth year. The age at diagnosis was calculated as the difference between the year of first diagnosis and the child’s year of birth. Children diagnosed with CP during their first year of life were classified as ‘within the first year of life’.

### Categorization of risk factors for CP and other variables

We selected 10 risk factors in this study based on the classification of risk factors presented in pediatric neurology textbooks.^15–17^ The selected risk factors were: (1) prematurity, (2) low birth weight, (3) birth-related conditions, (4) brain anomalies, (5) metabolic diseases, (6) infections, (7) brain infections, (8) brain injuries, (9) cerebrovascular disease, and (10) encephalopathy. All ICD-10 codes used to define the risk factors are listed in Supplementary Table S1. The risk factors were defined as follows: prematurity, birth before 37 weeks of gestation; low birth weight, birth weight lower than 2,500 g; asphyxia/hypoxia, a clinical condition characterized by insufficient oxygen supply during or immediately after birth, often defined by low Apgar scores or need for resuscitation; traumatic brain injury due to birth injury, physical trauma to the neonate’s head during labor/delivery; non-traumatic brain hemorrhage/hypoxic–ischemic encephalopathy, intracranial hemorrhage or global brain injury caused by hypoxia–ischemia in the perinatal period, typically diagnosed via neuroimaging and clinical signs; brain anomaly, congenital malformations of brain structure; hypoglycemia, blood glucose level <45 mg/dL in the neonatal period; hyperbilirubinemia, elevated serum bilirubin levels in neonates, which when severe, may progress to kernicterus; bacterial sepsis, systemic infection with positive blood culture in the neonatal period; meningitis/encephalitis, inflammation of the meninges or brain parenchyma due to infection; abscess, a focal, pus-containing infection of the intracranial or intraspinal space; traumatic brain injury, injury to the brain resulting from external mechanical force postnatally; cerebrovascular disease, an acute cerebrovascular event–intracranial hemorrhage or ischemic stroke–or its sequelae occurring during the postnatal period; encephalopathy, global brain dysfunction due to various systemic or toxic insults. Only risk factors diagnosed before or during the same year as the initial CP diagnosis were included. If a patient had more than one risk factor, each was counted separately.

South Korea’s administrative divisions consist of the capital city of Seoul, six metropolitan municipalities, and eight provincial regions. We categorized residential areas into three groups based on these administrative status and urban scale: capital city (Seoul), regional metropolitan cities (Busan, Daegu, Incheon, Gwangju, Daejeon, and Ulsan), and provincial regions (other regions).^18^ Seoul, the capital and largest city of South Korea with a population of approximately 10 million, has distinct administrative and urban characteristics that warrant separate classification. Regional metropolitan cities share similar administrative status (first-tier local governments defined by the Korean Local Autonomy Act) and urban characteristics, whereas the remaining regions consist of provincial cities, small towns, and rural counties

### Statistical analysis

Student’s t-test or Chi-squared test was used to compare patient characteristics. The primary incidence analyses involved calculating crude and age- and sex-standardized incidence rates by year of CP diagnosis. Crude incidence rates were determined by dividing the annual number of CP cases among children aged 5 years or younger by the corresponding mid-year population estimates, which were obtained from the Korean Statistical Information Service. Age- and sex- standardized incidence rates were calculated using the direct standardization method, with the 2010 mid-year population of Korean children aged 5 years or younger serving as the standard population.

Sex-specific incidence rates were calculated by dividing the number of CP cases in males or females by the respective mid-year sex-specific population for each year. Age-standardization of sex-specific rates was performed using the 2010 male and female Korean mid-year populations as standard references.

Age-specific incidence rates by age at diagnosis (within the first year of life, 1, 2, 3, 4, and 5 years of age) were computed by dividing the number of CP cases diagnosed at each specific age in a given year by the mid-year population for that age.

Additionally, the cumulative incidence of CP at five-year follow-up was analyzed by birth cohort from 2005 to 2020. Cumulative incidence was calculated by dividing the number of CP cases diagnosed within five years of birth in each birth cohort by the total number of live births in that birth cohort.

Joinpoint regression analysis was used to evaluate temporal trends in CP incidence.^19^ The annual percentage change (APC) and average annual percentage change (AAPC) were calculated for incidence rates over the study period. All statistical analyses were conducted using SAS version 9.4 (SAS Institute, Cary, NC). Joinpoint analysis was performed using the Joinpoint Regression Program version 4.9.0.0 (National Cancer Institute, Bethesda, MD). All analyses were two-sided, and statistical significance was defined at *P* < 0.05.

## Results

The classification of risk factors for pediatric CP is presented in Supplementary Table S1, and the general characteristics of the study population according to the period of CP diagnosis are shown in Table 1. The mean age at diagnosis was 1.44 years in 2005–2014 and 1.76 years in 2015–2024. There were no significant sex differences between the two periods. Regarding patient residence, the proportion of children living in capital city decreased (18.85% in 2005–2014 and 14.82% in 2015–2024), whereas those living in provincial regions increased (56.54% in 2005–2014 and 61.57% in 2015–2024). In both periods, tertiary general hospitals accounted for the highest proportion of initial CP diagnoses (45.39% in 2005–2014 and 47.68% in 2015–2024), followed by general hospitals (34.39% and 33.80%, respectively).

**Table 1:** General characteristics of patients by the period of cerebral palsy diagnosis.

	2005‒2014	2015‒2024	*p*-value^*a*^
*n* (%)	16,920 (100)	9,151 (100)	
Mean age at CP diagnosis (mean, SD)	1.44 (1.26)	1.76 (1.32)	<0.001
Sex, *n* (%)			
Male	9,735 (57.54)	5,257 (57.45)	0.890
Female	7,185 (42.46)	3,894 (42.55)	
Residence, *n* (%)			
Capital city	3190 (18.85)	1356 (14.82)	<0.001
Regional metropolitan cities	4068 (24.04)	2158 (23.58)	
Provincial regions	9566 (56.54)	5634 (61.57)	
Missing	96 (0.57)	3 (0.03)	
Type of hospital that diagnosed cerebral palsy, *n* (%)			
Tertiary general hospital	7,680 (45.39)	4,363 (47.68)	<0.001
General hospital	5,818 (34.39)	3,093 (33.80)	
Hospital	1,905 (11.26)	647 (7.07)	
Clinic	1,189 (7.03)	749 (8.18)	
Others	323 (1.91)	299 (3.27)	
Missing	5 (0.03)	0 (0)	

^a^*P*-value for student’s *t*-test of chi-squared test.

*CP* cerebral palsy, *SD* standard deviation.

Table 2 presents the distribution of risk factors according to the age at CP diagnosis. Prematurity and asphyxia/hypoxia were the most prevalent conditions among children diagnosed with an ICD-10 code for CP under 2 years of age (prematurity: 44.43% within the first year of life, 41.35% at age 1 year; asphyxia/hypoxia: 44.12% within the first year of life, 40.69% at age 1 year). From age 2 years onward, the proportions of prematurity, asphyxia/hypoxia, and hyperbilirubinemia became comparable (34.16%, 33.11%, and 33.55%, respectively). Among those diagnosed at ages 3–5 years, hyperbilirubinemia was the most common risk factor (31.24% at age 3 years, 28.64% at age 4 years, and 24.81% at age 5 years).

**Table 2:** Distribution of the risk factors of cerebral palsy in patients by age at diagnosis of pediatric cerebral palsy.

	Within the first year of life	1 year old	2 years old	3 years old	4 years old	5years old	*p*-value^*a*^
*n* (%)	4832 (100)	10,797 (100)	5243 (100)	2503 (100)	1648 (100)	1048 (100)	
Risk factors							
Prematurity, *n* (%)							
Yes	2147 (44.43)	4465 (41.35)	1791 (34.15)	677 (27.04)	315 (19.11)	155 (14.79)	<0.001
No	2685 (55.56)	6332 (58.64)	3452 (65.84)	1826 (72.95)	1333 (80.88)	893 (85.2)	
Low birth weight, *n* (%)							
Yes	1828 (37.83)	3880 (35.93)	1523 (29.04)	589 (23.53)	269 (16.32)	142 (13.54)	<0.001
No	3004 (62.16)	6917 (64.06)	3720 (70.95)	1914 (76.46)	1379 (83.67)	906 (86.45)	
Asphyxia/hypoxia, *n* (%)							
Yes	2132 (44.12)	4393 (40.68)	1736 (33.11)	653 (26.08)	316 (19.17)	151 (14.4)	<0.001
No	2700 (55.87)	6404 (59.31)	3507 (66.88)	1850 (73.91)	1332 (80.82)	897 (85.59)	
Traumatic brain injury due to birth injury, *n* (%)							
Yes	115 (2.37)	169 (1.56)	55 (1.04)	21 (0.83)	12 (0.72)	8 (0.76)	<0.001
No	4717 (97.62)	10628 (98.43)	5188 (98.95)	2482 (99.16)	1636 (99.27)	1040 (99.23)	
Non-traumatic brain hemorrhage /hypoxic ischemic encephalopathy, *n* (%)							
Yes	1302 (26.94)	2584 (23.93)	1065 (20.31)	371 (14.82)	192 (11.65)	90 (8.58)	<0.001
No	3530 (73.05)	8213 (76.06)	4178 (79.68)	2132 (85.17)	1456 (88.34)	958 (91.41)	
Brain anomaly, *n* (%)							
Yes	449 (9.29)	1273 (11.79)	689 (13.14)	307 (12.26)	181 (10.98)	134 (12.78)	<0.001
No	4383 (90.7)	9524 (88.2)	4554 (86.85)	2196 (87.73)	1467 (89.01)	914 (87.21)	
Hypoglycemia, *n* (%)							
Yes	386 (7.98)	840 (7.77)	404 (7.7)	199 (7.95)	121 (7.34)	91 (8.68)	0.853
No	4446 (92.01)	9957 (92.22)	4839 (92.29)	2304 (92.04)	1527 (92.65)	957 (91.31)	
Hyperbilirubinemia, *n* (%)							
Yes	1943 (40.21)	4067 (37.66)	1759 (33.54)	782 (31.24)	472 (28.64)	260 (24.8)	<0.001
No	2889 (59.78)	6730 (62.33)	3484 (66.45)	1721 (68.75)	1176 (71.35)	788 (75.19)	
Bacteria sepsis, *n* (%)							
Yes	1195 (24.73)	2577 (23.86)	1090 (20.78)	475 (18.97)	289 (17.53)	178 (16.98)	<0.001
No	3637 (75.26)	8220 (76.13)	4153 (79.21)	2028 (81.02)	1359 (82.46)	870 (83.01)	
Meningitis, *n* (%)							
Yes	136 (2.81)	356 (3.29)	196 (3.73)	102 (4.07)	75 (4.55)	51 (4.86)	<0.001
No	4696 (97.18)	10441 (96.7)	5047 (96.26)	2401 (95.92)	1573 (95.44)	997 (95.13)	
Encephalitis, *n* (%)							
Yes	68 (1.4)	185 (1.71)	137 (2.61)	77 (3.07)	52 (3.15)	39 (3.72)	<0.001
No	4764 (98.59)	10612 (98.28)	5106 (97.38)	2426 (96.92)	1596 (96.84)	1009 (96.27)	
Abscess, *n* (%)							
Yes	9 (0.18)	33 (0.3)	14 (0.26)	5 (0.19)	4 (0.24)	3 (0.28)	0.809
No	4823 (99.81)	10764 (99.69)	5229 (99.73)	2498 (99.8)	1644 (99.75)	1045 (99.71)	
Viral infection of CNS, *n* (%)							
Yes	7 (0.14)	19 (0.17)	17 (0.32)	10 (0.39)	6 (0.36)	4 (0.38)	0.082
No	4825 (99.85)	10778 (99.82)	5226 (99.67)	2493 (99.6)	1642 (99.63)	1044 (99.61)	
Bacterial infection of CNS, *n* (%)							
Yes	0 (0)	0 (0)	2 (0.03)	0 (0)	1 (0.06)	0 (0)	0.112
No	4832 (100)	10797 (100)	5241 (99.96)	2503 (100)	1647 (99.93)	1048 (100)	
Cysticercosis, *n* (%)							
Yes	0 (0)	0 (0)	0 (0)	0 (0)	0 (0)	0 (0)	-
No	4832 (100)	10797 (100)	5243 (100)	2503 (100)	1648 (100)	1048 (100)	
Traumatic brain injury, *n* (%)							
Yes	131 (2.71)	493 (4.56)	365 (6.96)	266 (10.62)	173 (10.49)	127 (12.11)	< 0.001
No	4701 (97.28)	10304 (95.43)	4878 (93.03)	2237 (89.37)	1475 (89.5)	921 (87.88)	
Intracranial hemorrhage/stroke, *n* (%)							
Yes	617 (12.76)	1252 (11.59)	564 (10.75)	225 (8.98)	166 (10.07)	78 (7.44)	<0.001
No	4215 (87.23)	9545 (88.4)	4679 (89.24)	2278 (91.01)	1482 (89.92)	970 (92.55)	
Toxic encephalopathy, *n* (%)							
Yes	1 (0.02)	2 (0.01)	0 (0)	1 (0.03)	1 (0.06)	0 (0)	0.660
No	4831 (99.97)	10795 (99.98)	5243 (100)	2502 (99.96)	1647 (99.93)	1048 (100)	
Anoxic brain damage, *n* (%)							
Yes	436 (9.02)	715 (6.62)	271 (5.16)	131 (5.23)	65 (3.94)	42 (4)	<0.001
No	4396 (90.97)	10082 (93.37)	4972 (94.83)	2372 (94.76)	1583 (96.05)	1006 (95.99)	
Unspecified encephalopathy, *n* (%)							
Yes	92 (1.9)	207 (1.91)	107 (2.04)	66 (2.63)	45 (2.73)	30 (2.86)	0.029
No	4740 (98.09)	10590 (98.08)	5136 (97.95)	2437 (97.36)	1603 (97.26)	1018 (97.13)	
Metabolic encephalopathy, *n* (%)							
Yes	3 (0.06)	9 (0.08)	10 (0.19)	1 (0.03)	0 (0)	1 (0.09)	0.137
No	4829 (99.93)	10788 (99.91)	5233 (99.8)	2502 (99.96)	1648 (100)	1047 (99.9)	

^a^*P*-value for chi-squared test.

*CNS* central nervous system.

**Fig. 1:**
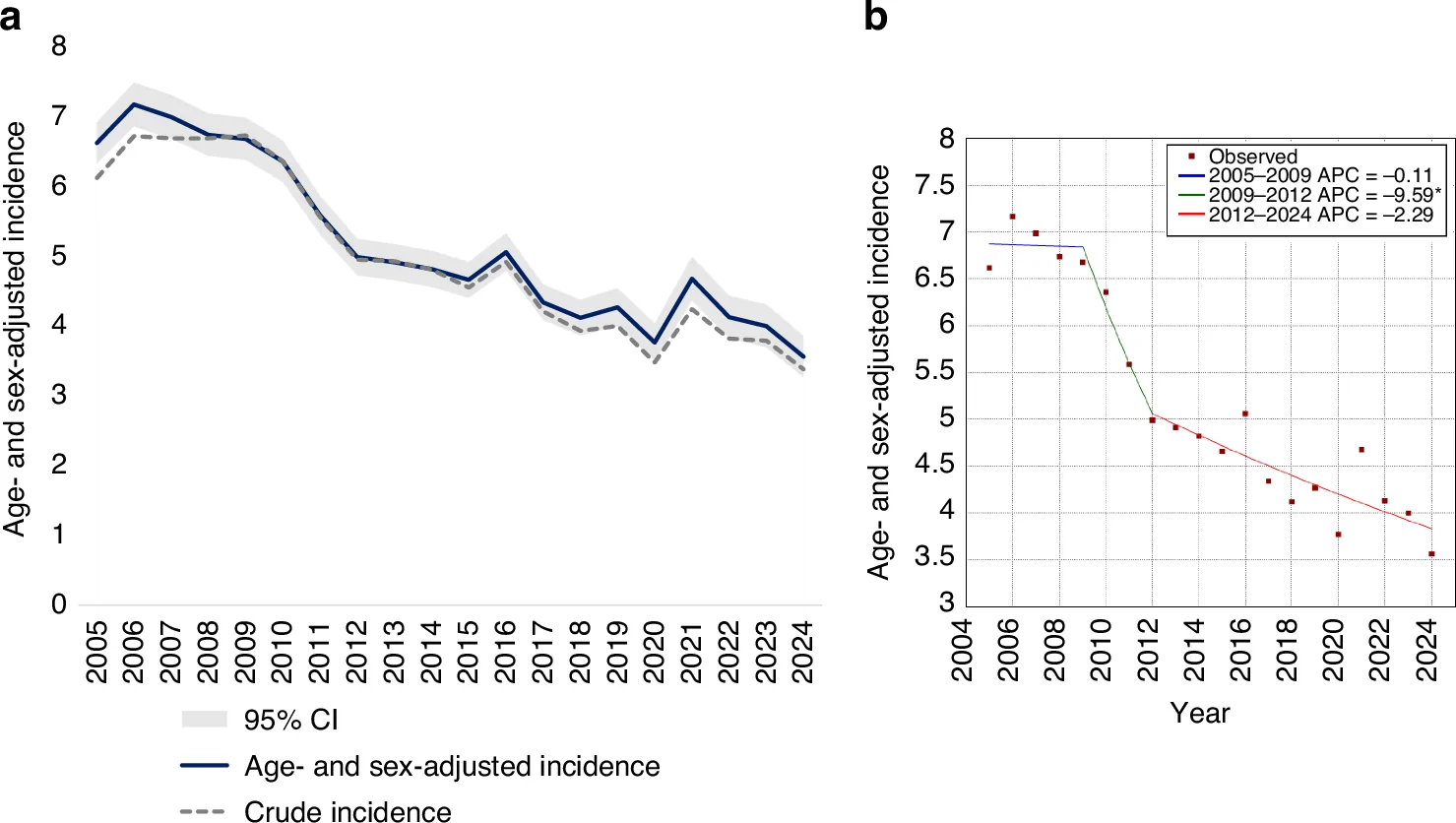
Annual incidence and annual percent change of cerebral palsy in children under 5 years of age in South Korea. **a** Annual incidence of pediatric cerebral palsy. **b** Joinpoint regression graph describing the annual percent change in incidence of pediatric cerebral palsy. * Indicates that the Annual Percent Change (APC) is significantly different from zero at the alpha = 0.05 level. APC annual percent change.

Figure 1 presents the annual incidence rates of CP under 5 years of age by year of diagnosis from 2005 to 2024. The crude incidence rate—calculated as the number of CP cases divided by the mid-year population of children under five—dropped from 6.12 per 10,000 in 2005 to 3.38 per 10,000 in 2024, reflecting an average annual percent change (AAPC) of −3.7% (P < 0.05). To adjust for demographic changes over time, age- and sex-standardized incidence rates were decreased from 6.62 per 10,000 in 2005 to 3.56 per 10,000 in 2024, with an AAPC of –3.0%.

**Fig. 2:**
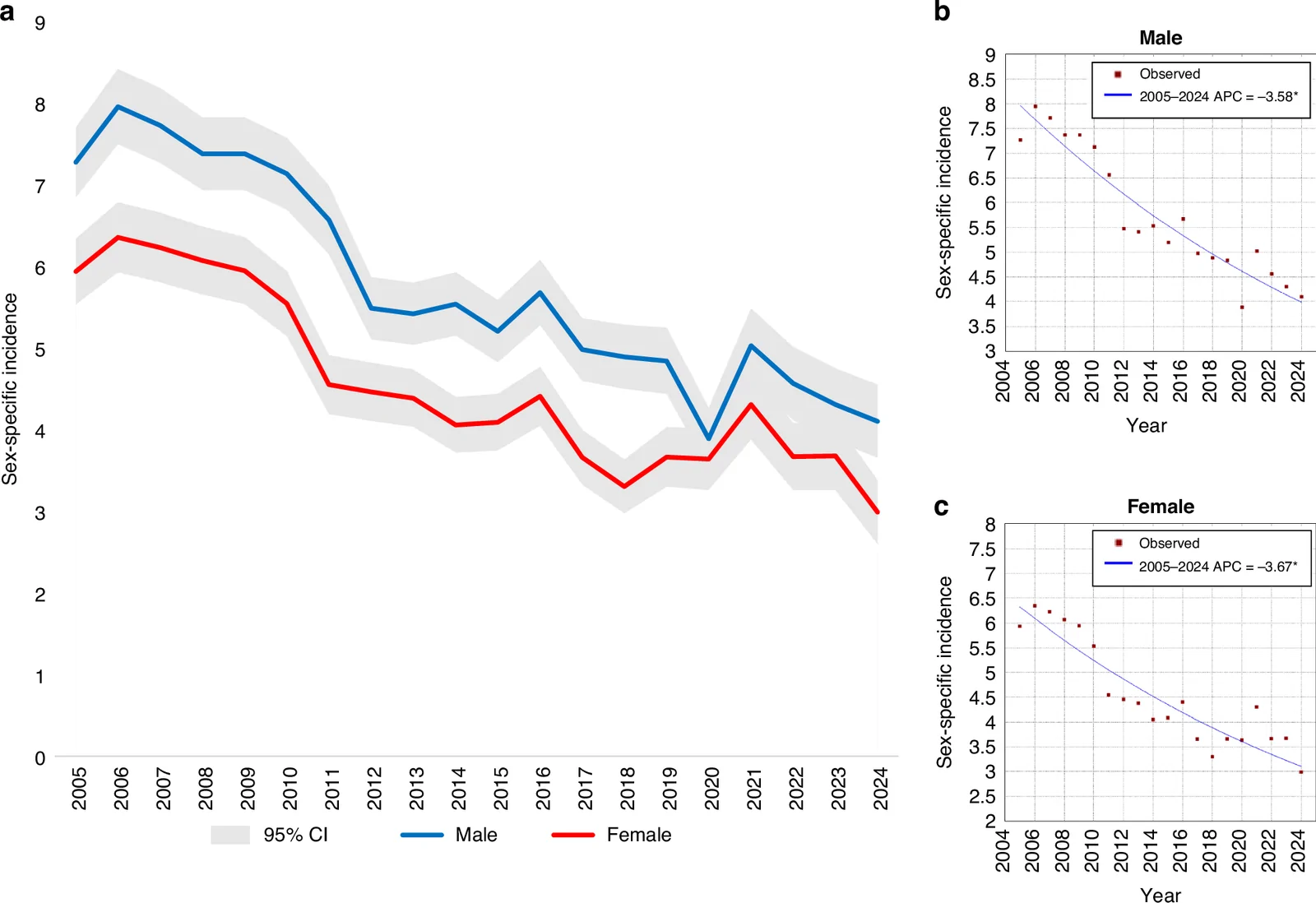
Annual incidence and annual percent change of cerebral palsy by sex. **a** Sex-specific incidence of cerebral palsy. **b** Annual percent change for males. **c** Annual percent change for females. APC annual percent change.

**Fig. 3:**
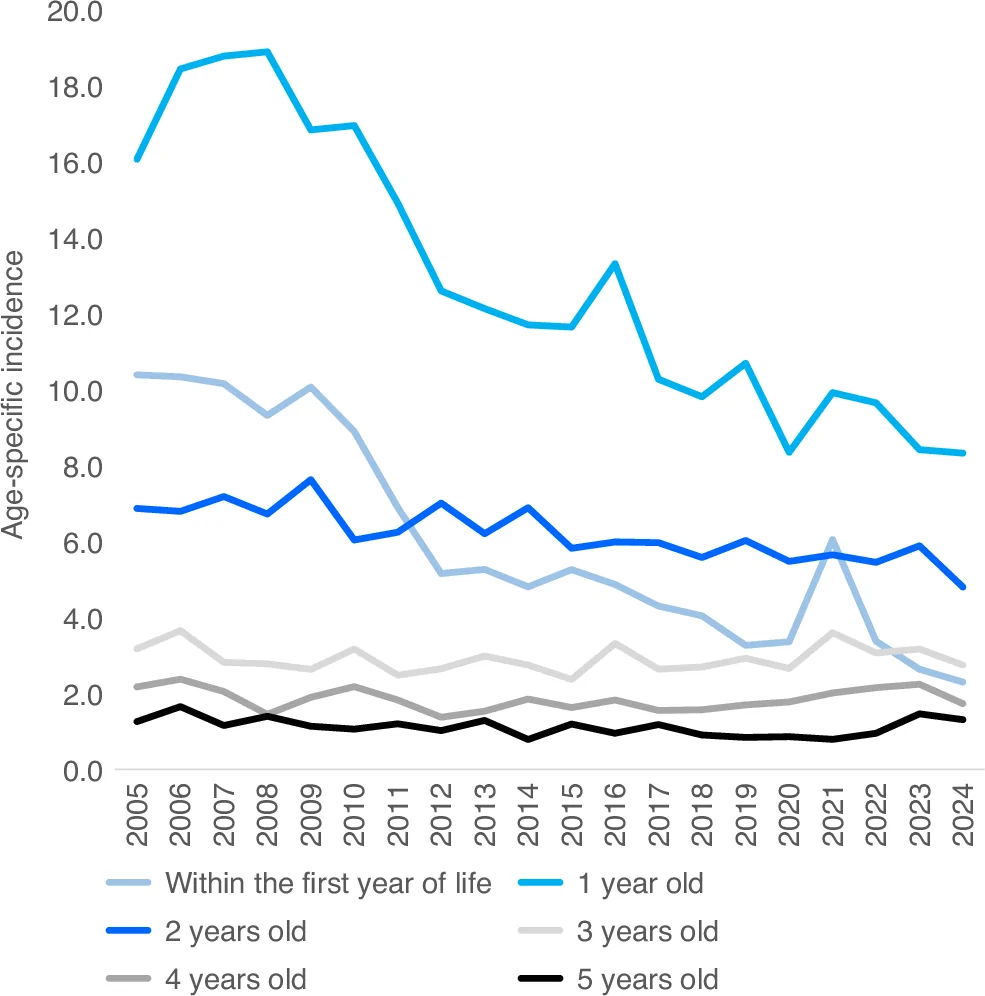
Age-specific incidence of cerebral palsy in children by age at diagnosis.

Sex-specific trends showed that incidence rates declined more rapidly among females than males (male AAPC = −3.58%, *P* < 0.05; female AAPC = −3.67%, *P* < 0.05; Fig. 2). As shown in Fig. 3, age-specific incidence rates were consistently highest at age 1 year throughout the study period. From 2005 to 2011, the incidence within the first year of life was higher than at age 2 years, but from 2012 onward, the rate at age 2 years surpassed that within the first year of life. The incidence rates for within the first year of life, ages 1, and 2 years declined significantly over the study period, with the steepest decrease observed within the first year of life (AAPC = −7.2%, *P* < 0.05), followed by age 1 year (AAPC = −3.9%, *P* < 0.05) and age 2 years (AAPC = −1.5%, P < 0.05; Supplementary Fig. S1).

The 5-year cumulative incidence of CP by birth cohort is summarized in Table 3. For infants born in 2005, the was 4.26 per 1000 live births, compared to 2.51 per 1000 live births for those born in 2020. From 2005 to 2020, the decreased significantly, with an AAPC of −3.5% per birth year (*P* < 0.05).

**Table 3:** Cumulative incidence of pediatric cerebral palsy by birth cohorts at 5 years old.

Birth Year	The No. of live birth	Total CP cases within 5 years of follow-up	Cumulative incidence within 5 years of follow-up (per 1000 live birth)
2005	438,707	1867	4.26
2006	451,759	1819	4.03
2007	496,822	1980	3.99
2008	465,892	1766	3.79
2009	444,849	1717	3.86
2010	470,171	1667	3.55
2011	471,265	1448	3.07
2012	484,550	1407	2.90
2013	436,455	1312	3.01
2014	435,435	1197	2.75
2015	438,420	1302	2.97
2016	406,243	1109	2.73
2017	357,771	988	2.76
2018	326,822	945	2.89
2019	302,676	757	2.50
2020	272,337	684	2.51
AAPC	-	-	−3.5^*^

^*^Indicates that the Annual Percent Change (AAPC) is significantly different from zero at the alpha=0.05 level.

*CP* cerebral palsy, *AAPC* average annual percent change.

**Fig. 4:**
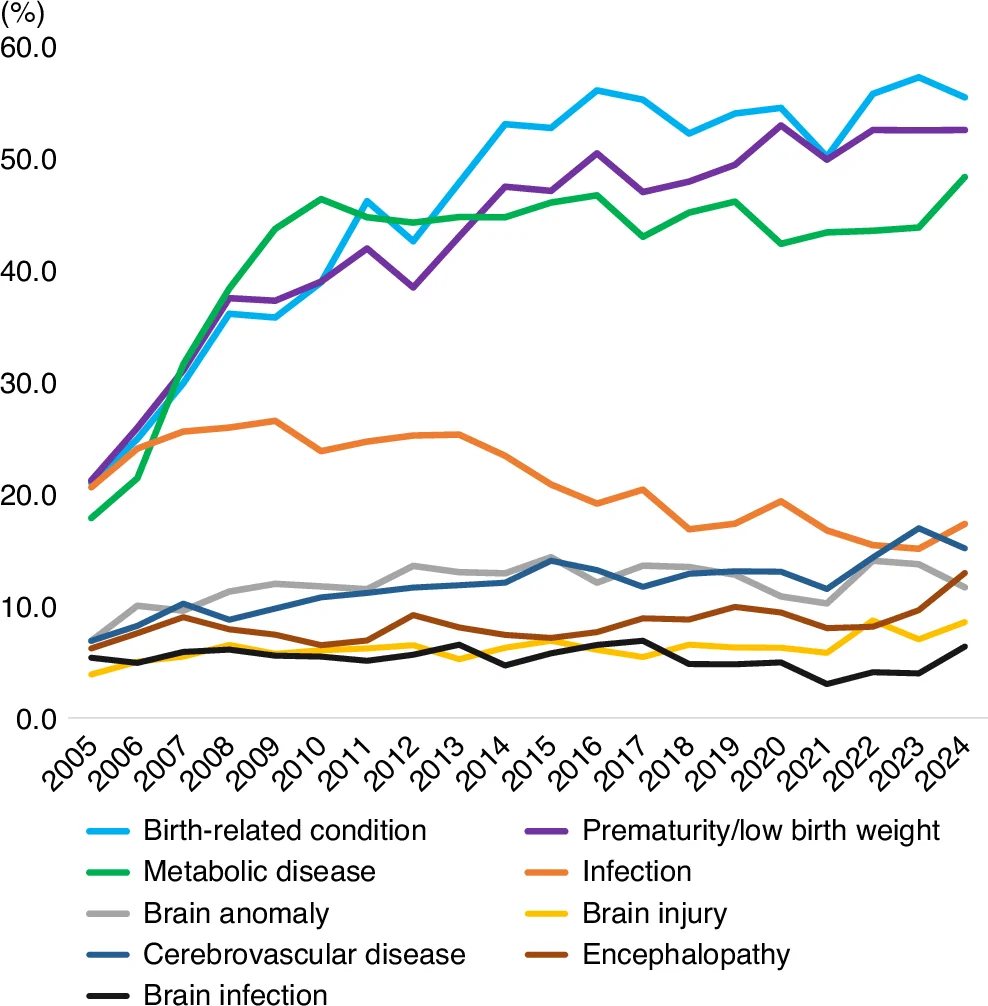
Distribution of risk factors in children with cerebral palsy.

Figure 4 displays the distribution of risk factors among patients with CP. The most prevalent risk factors were birth-related complications, prematurity/low birth weight, and metabolic diseases, all of which showed increasing trends over time (birth-related conditions: AAPC = 5.2%, *P* < 0.05; prematurity/low birth weight: AAPC = 4.9%, *P* < 0.05; metabolic diseases: AAPC = 4.8%, *P* < 0.05).

## Discussion

In this population-based retrospective cohort study, we found that the age- and sex- adjusted incidence of CP among children aged 5 years or younger in South Korea steadily decreased from 6.62 per 10,000 children in 2005 to 3.56 per 10,000 children in 2024, representing an average annual decline of 3%. The sex-specific incidence was higher in male, and the age-specific incidence was the highest at age 1 year. The most prevalent risk factors were birth-related complications, prematurity/low birth weight, and metabolic diseases, all of which showed increasing trends over time. The 5-year cumulative incidence of CP steadily decreased from 4.26 per 1,000 live births in the 2005 birth cohort to 2.51 per 1,000 in the 2020 birth cohort, reflecting an average annual decline of 3.5%.

Over the past two decades, the age- and sex- standardized incidence of CP in South Korea decreased by approximately 50%, from 7.17 per 10,000 children in 2006 to 3.56 per 10,000 in 2024. This result is consistent with previous studies in Korea^10,11^ and with those of other high-income countries that have reported a decrease in CP prevalence.^20,21^ The number of low birth weight and premature infants who are considered high-risk groups for CP has increased sharply in South Korea. In South Korea, the preterm birth rate—babies born at 36 weeks or earlier—increased from 47.3 per 1000 live births in 2005 to 97.8 per 1000 live births in 2023.^22^ Similarly, the low birth weight rate—babies weighing less than 2.5 kilograms at birth—rose from 42.6 per 1000 live births in 2005 to 76.2 per 1000 live births in 2023.^23^ However, the development of neonatal intensive care has enabled many medically fragile newborns to survive,^24^ leading to an increase in the temporary occurrence of CP until 2006, which is similar in other countries.^25,26^ The occurrence of CP often regarded as an indicator of the overall quality of obstetric and neonatal care.^6^ In this context, despite a substantial rise in the number of premature and low birth weight infants over the study period, the incidence of CP has continued to decline. These observations are consistent with improvements in perinatal and neonatal care helping to mitigate an otherwise expected rise in CP incidence in Korea. Several system- and therapy-level advances plausibly contributed to this decline: (1) administration of antenatal magnesium sulfate for threatened preterm birth reduces the risk of CP and the composite outcome of death or CP and is now recommended by the World Health Organization and supported by updated Cochrane evidence^27^; (2) therapeutic hypothermia for moderate-severe hypoxic–ischemic encephalopathy lowers the probability of death or major neurodevelopmental disability^28^; (3) regionalization/expansion and quality gains in neonatal intensive care in Korea (capacity, staffing, organized transfers)^29–31^; (4) Group B streptococcus screening with intrapartum antibiotic prophylaxis reduces early-onset neonatal sepsis, a recognized pathway to brain injury^32^; and (5) standardized hyperbilirubinemia management prevents extreme hyperbilirubinemia and kernicterus phenotypes linked to dyskinetic CP.^33^ Collectively, these advances provide a biologically and systemically plausible explanation for the observed approximate 50% reduction despite rising prematurity.

In our study, the annual sex- and age-adjusted incidence of CP in South Korea decreased from 6.62 per 10,000 children in 2005 to 3.56 per 10,000 children in 2024. According to a nationwide cohort study conducted in Türkiye, the annual incidence of CP declined from 11 per 10,000 children (1.05 per 1,000) in 2016 to 4.7 per 10,000 children (0.47 per 1,000) in 2022.^34^ In contrast, a study using Kazakhstan’s national database reported an increase in the annual CP incidence from 4.5 per 10,000 children (44.9 per 100,000) in 2006 to 8.3 per 10,000 children (82.9 per 100,000) in 2016.^35^ Compared to these countries, the annual incidence of CP in South Korea was lower and demonstrated a significant decreasing trend over time.

In CP epidemiology, the “birth prevalence at age 5 years” commonly reported by European and Australian registries is conceptually similar to the 5-year cumulative incidence in this study. Because CP has a baseline prevalence of zero at birth, is generally irreversible, and rarely results in early mortality, the number of cumulative incident cases observed over 5 years in a birth cohort is expected to closely approximate the number of prevalent cases measured at age 5 years. Therefore, we compared the present 5-year cumulative incidence of CP by birth cohort with the birth prevalence reported at age 5 years in international studies. In this study, the 5-year cumulative incidence declined from 4.26 per 1,000 live births (birth year 2005) to 2.51 per 1,000 (birth year 2020). Multi-site European registry data likewise reported a decline in the CP birth prevalence from 1.90 to 1.77 per 1,000 live births across birth years 1980–2003, corresponding to a mean annual fall of about 0.7%.^21^ Australia also documented significant reductions in birth prevalence for birth years 1995–2009, including decreases among both extremely preterm and term infants, with a concurrent fall in moderate-to-severe disability.^20^ A recent global analysis estimated a pooled birth prevalence of approximately 1.6 per 1,000 in high-income countries.^8^ Taken together, South Korea’s absolute rates fall within the internationally reported range, and the pace of decline (AAPC − 3.5%) appears at least as steep as—and in some comparisons steeper than—those reported elsewhere, although differences in ascertainment windows and methods should be kept in mind when interpreting cross-country comparisons.

More than half of all patients with CP were male, and there was no difference in the sex ratio, which was consistent with those of previous studies that found a higher percentage of male compared to females among patients with CP.^26,36,37^ While the incidence of CP showed a slightly greater decline among females compared to males, the overall difference was minimal. (Fig. 2). This consistent male predominance, alongside the minimal difference in incidence trends between sexes, suggests that the sex distribution of CP has remained stable over time, despite the overall decline in incidence.

CP is usually diagnosed before the age of 5 years; however, it can now be diagnosed as early as 6 months of age using tests such as magnetic resonance imaging.^1,5,38^ In our study, more than half of the patients with CP were diagnosed under 2 years of age. In 2015–2024, the proportion of patients diagnosed under 2 years of age has declined compared to that in 2005–2014. However, the proportion of patients diagnosed at the age of 2–5 years has increased. However, this was only the result of a large reduction in incidence of CP diagnosed within the first year of life or 1 year of age, whereas the decrease in incidence of CP at age 2 years was relatively small and the incidence among children aged 3 to 5 years remained largely unchanged (Fig. 3). A decline in pre-/perinatal CP is a global trend.^8^ The decrease in the diagnosis rate within the first year of life is likely due to advances in neonatal intensive care. As noted above, while the survival rate of high-risk neonates has increased, the proportion of infants presenting with clinical symptoms severe enough for early CP diagnosis may have declined. This may explain the substantial decrease in CP diagnoses within the first year of life. Regarding the decrease in CP incidence at ages 2 to 5 years compared to under 2 years of age, a previous study reported that term-born children are diagnosed with CP later than preterm children, largely due to the absence of routine developmental checkups in those without identifiable risk factors.^39^ In our study, patients diagnosed between ages 2 and 5 years were more likely to lack identifiable risk factors and less likely to have a history of prematurity or low birth weight than those diagnosed under 2 years of age.

Nevertheless, the situation in Korea appears to differ from that in other countries. It is likely that the strong implementation of routine developmental checkups has facilitated earlier diagnoses of children with CP who, in the absence of clear risk factors or overt symptoms, might otherwise have remained undiagnosed or been diagnosed much later, potentially even after the age of 5 years. The National Health Screening Program for Infants and Children introduced in November 2007, is a nationwide health checkup initiative that periodically monitors children’s growth and development.^40^ As part of this program, the Korean Developmental Screening Test for Infants and Children (K-DST) is used to assess developmental progress from 4 to 71 months of age, regardless of underlying illness, enabling timely intervention when necessary.

Although the initial adoption the rate of K-DST was low, and it originally employed a slightly different tool, the revised version, following standardization and revalidation—is now widely implemented and recognized as an effective tool for assessing motor function.^40^ Through the K-DST, children with CP—particularly those without obvious risk factors or with milder symptoms who might otherwise have been missed—are increasingly being diagnosed earlier. Notably, although the overall proportion of CP diagnoses decreased with age after infancy, a greater proportion of diagnoses occurred between ages 2 and 5 years during 2015–2024 compared to 2005–2014. This shift may reflect the expanded reach and improved effectiveness of the K-DST in identifying children who previously would have been overlooked.

The fact that more than 50% of CP cases were diagnosed under 2 years of age is a positive outcome, as early diagnosis and intervention are critical for optimizing motor and cognitive plasticity, preventing secondary complications, and improving caregiver well-being.^38^ While promoting even earlier identification remains important, minimizing the harm associated with missed or delayed diagnoses is equally critical. In this context, the increased rate of diagnoses at ages 2 to 5 years may represent meaningful progress in childhood developmental surveillance in South Korea.

In our study, the diagnosis rate of CP was higher at age 1 year than within the first year of life. This may partly reflect the tendency for more apparent motor developmental delays to emerge after 12 months of age. Although the K-DST has proven useful for ^40^identifying CP, its questionnaire-based format likely yields higher detection accuracy in older children, as developmental delays become more pronounced and easier for caregivers to recognize and report.

In addition to CP diagnosis methods, it is also important to recognize that social systems and the attitudes of guardians toward the condition may influence diagnosis timing. Consequently, many children may have been receiving rehabilitation treatment without a formal diagnosis of CP. Measures should be taken to ensure these patients receive accurate CP diagnoses and appropriate treatment tailored to their needs.

In this study, the proportion of patients with CP with pre-/perinatal risk factors only has gradually increased and has been the most common risk factors of CP since 2007, accounting for approximately 60% of cases; if we include patients with both pre-/perinatal and postnatal risk factors, approximately 70–80% of patients with CP have pre-/perinatal risk factors, which is consistent with a previous study showing that the majority of patients with CP have pre-/perinatal risk factors.^6^ Patients with pre-/perinatal risk factors often have multiple risk factors rather than a single factor. The proportion of patients with postnatal risk factors alone remained unchanged at less than 5–10%; however, as the proportion of patients with both pre-/perinatal and postnatal risk factors gradually increased, the proportion of patients with postnatal causes gradually increased to approximately 20–30%, which was similar to previous studies that suggested that postnatal risk factors constitutes 10–25% of CP cases.^8,41,42^ Among the postnatal risk factors, the proportion of patients with cerebrovascular disease gradually increased but was not as high as previously reported.^43^ The proportion of patients with unclassified risk factors in our study has declined and remained below 20% since 2009. While this proportion is similar to those reported in previous studies,^37,43–45^ direct comparisons should be interpreted with caution, as the criteria for classifying cases as “unidentified” or “unclassified” vary across studies. In our study, some of these patients classified as having unclassified risk factors may have had prenatal risk factors that could not be detected due to limitations in the available data. Furthermore, recent studies have reported that genetic variants are present in 31–55% of patients with CP of unidentified causes.^45–48^ Based on these findings, it is plausible that a considerable proportion of our patients with unclassified risk factors may have underlying genetic variants. Therefore, for patients exhibiting clinical features of CP without an identifiable risk factor, genetic testing could play a crucial role in facilitating early diagnosis and timely intervention.

This study has several limitations. Owing to the nature of insurance claims data, misclassification bias can occur if the diagnosis is not entered correctly. In the past, the proportion of patients with an unclassified risk factor was much higher than that reported in other studies, possibly because of missing data on the underlying disease. However, in the data from 2009 onward, there was no significant difference in the rate of causal disease compared with other studies, which is likely a reliable result. The data did not allow us to see the results of tests, such as genetic tests. Some patients with unclassified risk factors may have had a confirmed genetic cause; however, we were unable to confirm this because of the unavailability of test results. Some patients may have symptoms of CP; however, owing to an abnormality in the associated genetic tests, they are given a genetic diagnosis instead of a diagnosis of CP, which may be missed in patients with CP. Finally, this study used healthcare utilization data for each patient, which made it difficult to obtain information on maternal factors and antenatal factors. Nevertheless, this study is significant in that it identified changes in the incidence of CP and its risk factors by analyzing long-term national data.

## Conclusion

The incidence of CP in South Korea decreased from 2006 to 2025. The age-specific incidence was the highest at age 1 year and the most prevalent risk factors were birth-related complications, prematurity/low birth weight, and metabolic diseases. We infer that advances in perinatal care may have contributed to the decline in CP incidence among children aged ≤2 years, and that the developmental screening test for infants and children implemented in Korea may have contributed milder-case detection, perhaps increasing the relative proportion diagnosed at ages 4–5 years. To sustain the decline in CP incidence, further efforts should focus on further preventing risk factors, and more effective implementation of developmental screening tests should be done to promote the detection of CP.

## Supplementary information

Supplementary Materials 3rdRv v1.2 AfterReturn

## Data Availability

This study is based on the Korean National Health Insurance Service Database (NHIS-2024-1-448). These data do not belong to the authors but to the Korean National Health Insurance Service, and the authors are not permitted to share them, except in aggregate form.
