# Supplementary material for: Trends in the annual incidence and risk factors of cerebral palsy
Source: Pediatr Res. 2026 Jan 23;100(3):1143–51. doi: 10.1038/s41390-025-04752-0 (PMC13672078; doi:10.1038/s41390-025-04752-0)
Supplement: Supplementary file 1 [file 41390_2025_4752_MOESM1_ESM.pdf]

SUPPLEMENTARY MATERIALS

Trends in the annual incidence and risk factors of cerebral palsy

CONTENTS

Supplementary Table S1 2

Supplementary Figure S1 6

TABLES

Supplementary Table S1. Diagnosis codes for the risk factors of pediatric cerebral palsy

Classifications	Definition	International Classification of Diseases 10th Revision
**i. Prematurity**	Birth before 37 weeks of gestation	P072*, P073*
**ii. Low birth weight**	Birth weight less than 2,500 grams	P070*, P071*
**iii. Birth-related condition**		
Asphyxia/hypoxia	A clinical condition characterized by insufficient oxygen supply during or immediately after birth, often defined by low Apgar scores or need for resuscitation	P20*, P21*, P22, P220*, P228*, P229*
Traumatic brain injury due to birth injury	Physical trauma to the neonate's head during labor/delivery	P10*, P110*, P111*, P112*
Non-traumatic brain hemorrhage /hypoxic ischemic encephalopathy	Intracranial hemorrhage or global brain injury from hypoxia-ischemia in the perinatal period, , typically diagnosed via neuroimaging and clinical signs	P52*, P91*

**iv. Brain anomaly**	Congenital malformations of brain structure	Q00*, Q01*, Q02*, Q03*, Q04*, Q07*
**v. Metabolic disease**		
Hypoglycemia	Blood glucose level < 45mg/dL in the neonatal period	E161*, E162*, P70*
Hyperbilirubinemia	Elevated serum bilirubin levels in neonates, when severe, may progress to kernicterus	P57*, P58*, P59*, R17*, E80*
**vi. Infection**		
Bacterial sepsis	Systemic infection with positive blood culture in the neonatal period	A021*, A207*, A227*, A241*, A267*, A327*, A40*, A41*, A427, A548*, B007*, B377, P36*, R651*, T802*, T814*, T880*
**vii. Brain infection**		
Meningitis	Inflammation of meninges due to infection	
Bacterial meningitis	-	A20.3, A32.1, A39.0, G00, G01
Tuberculosis meningitis	-	A17.0, A17.81
Viral meningitis	-	B00.3, B01.0, B05.1, B26.1, A87, G02.0

Fungal meningitis	-	B37.5, B38.4, B45.1, G02.1
Parasites	-	G02.8
Unspecified cause	-	G04.0, G04.8, G04.9, G05.8
Encephalitis	Inflammation of brain parenchyma due to infection	
Bacterial encephalitis	-	G04.2, G05.0
Viral encephalitis	-	B00.4, B01.1, B05.0, B26.2, A86, A81.1, A83*, A84*, A85*, A86*, G05.1
Parasites	-	G04.0, G04.8, G04.9, G05.8
Unspecified cause	-	G05.2
Abscess	A focal, pus-containing infection of the intracranial or intraspinal space	G06*, G07
Viral infection of central nervous system	-	A88*, A89*, A81.8, A81.9
Bacterial infection of central nervous system	-	A52.3
Cysticercosis	-	B69.0
**viii. Brain injury**		

Traumatic brain injury	Injury to the brain resulting from external mechanical force postnatally	S06*
**ix. Cerebrovascular disease**	An acute cerebrovascular event or its sequelae during the postnatal period	
Intracranial hemorrhage /Intraventricular hemorrhage/ Ischemic stroke	-	I60*–I69*
**x. Encephalopathy**	Global brain dysfunction due to various systemic or toxic insults	
Toxic encephalopathy	-	G92*
Anoxic brain damage	-	G931*
Unspecified encephalopathy	-	G934*
Metabolic encephalopathy	-	G9380*

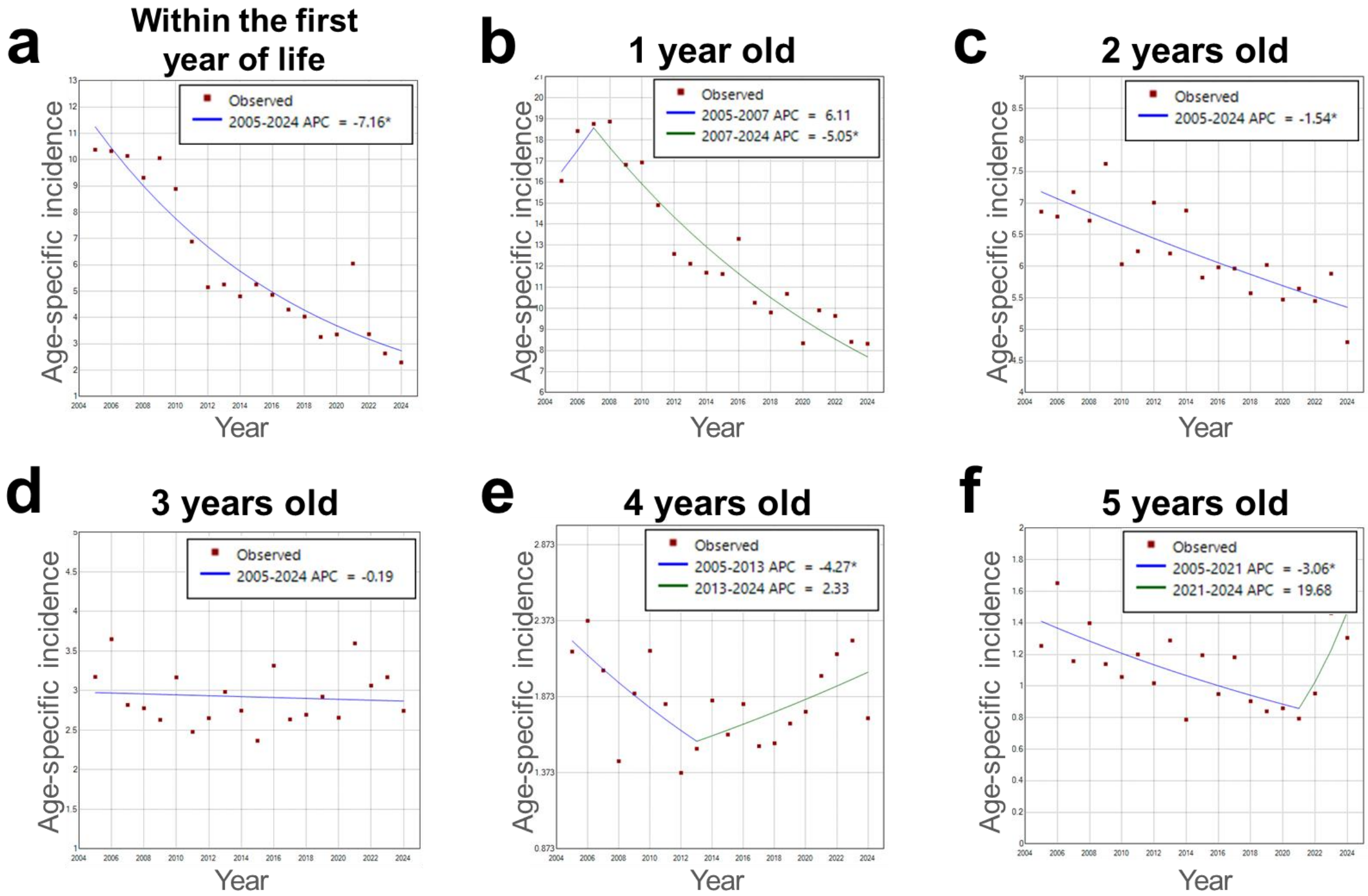

Supplementary Figure S1. The annual percent change of cerebral palsy in children by age at diagnosis. (a) within the first year of life, (b) 1 year old, (c) 2 years old, (d) 3 years old, (e) 4 years old, (f) 5 years old.

APC, annual percent change
